# Lactate dehydrogenase is a prognostic indicator in patients with hepatocellular carcinoma treated by sorafenib: results from the real life practice in HBV endemic area

**DOI:** 10.18632/oncotarget.13428

**Published:** 2016-11-17

**Authors:** Mu-xing Li, Hong Zhao, Xin-yu Bi, Zhi-yu Li, Xue-song Yao, Huai Li, Zhen Huang, Yue Han, Jian-guo Zhou, Jian-jun Zhao, Ye-fan Zhang, Dong-bin Zhao, Jian-qiang Cai

**Affiliations:** ^1^ Department of Abdominal Surgical Oncology, National Cancer Center/Cancer Hospital, Chinese Academy of Medical Sciences and Peking Union Medical College (CAMS & PUMC), Beijing 100021, P. R. China; ^2^ Department of Interventional Therapies, National Cancer Center/Cancer Hospital, Chinese Academy of Medical Sciences and Peking Union Medical College (CAMS & PUMC), Beijing 100021, P. R. China

**Keywords:** LDH, hepatocellular carcinoma, sorafenib, prognosis, HBV endemic area

## Abstract

**Purpose:**

Lactate dehydrogenase (LDH), which was an indirect marker of hypoxia, was a potentially prognostic factor in several malignancies. There is a lack of evidence about the prognostic value of serum LDH level in patients with hepatocellular carcinoma (HCC) receiving sorafenib treatment from hepatitis B virus endemic areas.

**Materials and Methods:**

A total of 119 HBV-related HCC patients treated by sorafenib from a Chinese center were included into the study. They were categorized into 2 groups according to the cut-off value of pre-treatment LDH, which was determined by the time dependent receiver operating characteristics (ROC) curve for the overall survival. The prognostic value of LDH was evaluated. The relationships between LDH and other clinicopathological factors were also assessed.

**Results:**

The cut-off value was 221 U/L. With a median follow up of 15 (range, 3-73) months, 91 patients reached the endpoint. Multivariate analysis proved that pre-treatment serum LDH level was an independent prognostic factor for both overall survival (OS) and progression-free survival (PFS). For patients whose pre-treatment LDH ≥ 221 U/L, increased LDH value after 3 months of sorafenib treatment predicted inferior OS and PFS. And patients with elevated pre-treatment LDH level predisposed to be featured with lower serum albumin, presence of macroscopic vascular invasion, advanced Child-Pugh class, advanced T category, higher AFP, and higher serum total bilirubin.

**Conclusions:**

Serum LDH level was a potentially prognostic factor in HCC patients treated by sorafenib in HBV endemic area. More relevant studies with reasonable study design are needed to further strengthen its prognostic value.

## INTRODUCTION

Hepatocellular carcinoma (HCC) ranks as the 5^th^ most common malignant cancer and the 3^rd^ most frequent cause of cancer leading death worldwide [1–3]. To our disappointment, a remarkable portion of the patients are not eligible for curative treatments including hepatectomy, radiofrequency ablation (RFA) and liver transplantation at their initial diagnosis [4]. Sorafenib, a multi-tyrosine kinase inhibitor, is the only Food and Drug Administration (FDA) approved molecular targeted therapy for the management of HCC at advanced stages [5]. However, heterogeneous clinical outcomes of patients treated by sorafenib have been observed. Some patients achieved long term disease control; while some patients were resistant to sorafenib treatment and suffered from unnecessary adverse effects at the meantime [6, 7]. Therefore it is essential to figure out a biomarker predicting the prognosis as well as guiding the selection of the candidates for sorafenib therapy [6, 8].

The interrelationship between hypoxia and tumor development has drawn a lot of attention in recent years. Lactate dehydrogenase (LDH), which is a key enzyme in the conversion of pyruvate to lactate under anaerobic environment [9], has been recognized as an indirect marker of the extent of tumor hypoxia, a key biological mechanism for the development of treatment resistance in cancer cells [10, 11]. Previous in-vitro studies revealed that hypoxia induced by sustained sorafenib treatment conferred tumor's resistance to sorafenib through hypoxia inducible factor-1α (HIF-1α) and nuclear factor kappa-light-chain-enhancer of activated B cells (NF-κB) activation [12]. Thus it is quite possible that serum LDH level may predict sorafenib therapeutic efficacy.

The prognostic value of LDH has been extensively studied in several types of malignant tumors including pancreatic cancer [13], colorectal cancer [14] as well as HCC [15]. Previous studies have unveiled the prognostic value of LDH in patients with HCC treated by hepatectomy [15] and transcatheter hepatic arterial chemoembolization (TACE) [16]. Compared with these studies, the tumor burden of patients taking sorafenib is much more severe. Meanwhile only 2 studies investigating the prognostic value of LDH have been published [17, 18]. Both of them were in sample size less than 100 and were conducted in Italy where the underlying etiology was predominantly hepatitis C virus (HCV). To the best of our knowledge, no studies evaluating the prognostic value of LDH in hepatitis B virus (HBV) endemic areas have been published yet. HBV-related HCC differed from HCV-related HCC quite a lot in terms of the pathogenesis and oncological features [19], which was also mentioned in the discussion of Oriental trial by Cheng et al. [20]. Therefore we felt it was essential and novel to perform the present retrospective analysis in order to investigate the prognostic value of serum LDH in HBV-related HCC patients.

## RESULTS

### The characteristics of the patients

**Figure 1:**
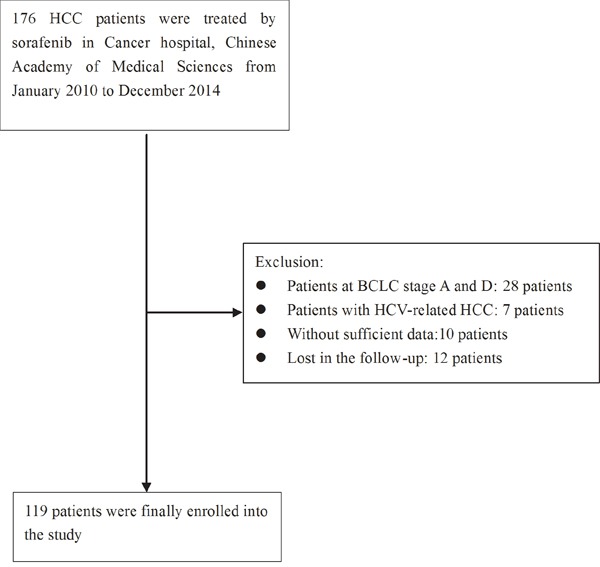
The flowchart describing the selection of the patients

The characteristics of the patients were summarized in Table 1. One hundred and six male patients and 13 female patients (Figure 1) with a median age of 54 (range 19-79) years old consisted the studied patients. Fifteen (15/119, 12.6%) patients were at BCLC stage B and 104 (104/119, 87.4%) patients were at BCLC stage C. Most patients (107/119, 89.9%) were at Child-Pugh class A. Macroscopic vascular invasion was detected in 47 (47/119, 39.5%) patients. Extrahepatic metastasis was detected in 67 (67/119, 56.3%) patients. One hundred and one (101/119, 84.9%) patients underwent treatments prior to the initiation of sorafenib treatment (Supplementary Table S1). Seventy two (72/119, 60.5%) patients underwent concomitant treatments after the initiation of sorafenib treatment (Supplementary Table S1). TACE was the most common therapy received, with 88 patients receiving TACE prior to sorafenib treatment and 67 patients receiving TACE concomitantly with sorafenib. The median duration of sorafenib treatment was 11 (range, 1–71) months. Stage 3-4 adverse events were observed in 45 (45/119, 37.8%) patients. Overall, 36 (36/119, 30.3%) patients experienced dose reduction during the treatment. A total of 31 (31/119, 26.1%) patients discontinued the sorafenib treatment. The main reasons for discontinuation of sorafenib were concurrent progression disease (PD) and (or) liver function deterioration (93.5%) and severe adverse events (6.5%).

**Table 1:** Clinicopathological features of the patients involved in this study

Variable	Value
Age (years)	54 (19-79) ^a^
Gender (Male/Female)	106/13
Albumin (g/L)	39.6±5.4 ^c^
Total Bilirubin (μmol/L)	22.2±15.9^c^
Prothrombin time (seconds)	12.1±1.1^c^
AFP (ng/ml)	188.3 (1.2-483307.0) ^a^
Pre-treatment LDH (U/L)	301.5±243.6^c^
Child-Pugh Class	
A	107 (89.9%)^b^
B	12 (10.1%)^b^
Tumor number	
Solitary	48 (40.3%)^b^
Multiple	71 (59.7%)^b^
Tumor size (cm)	7.2±4.7^c^
Macrovascular invasion	
Absent	72 (60.5%)^b^
Present	47 (39.5%)^b^
ECOG PS	
0	108 (90.8%)^b^
1	11 (9.2%)^b^
T category	
1	30 (25.2%) ^b^
2	18 (15.1%) ^b^
3	69 (58.0%) ^b^
4	2 (1.7%) ^b^
N category	
0	81 (68.1%) ^b^
1	38 (31.9%) ^b^
M category	
0	52 (43.7%)^b^
1	67 (56.3%)^b^
TNM	
II	11 (9.2%) ^b^
III	36 (30.3%) ^b^
IV	72 (60.5%) ^b^
BCLC stage	
B	15 (12.6%)^b^
C	104 (87.4%)^b^
Previous treatments	
Absent	18 (15.1%) ^b^
Present	101 (84.9%) ^b^
Concomitant treatments	
Absent	47 (39.5%) ^b^
Present	72 (60.5%) ^b^
Duration of sorafenib treatment (months)	11 (1-71) ^a^
Discontinuation	
Absent	88 (73.9%) ^b^
Present	31 (26.1%) ^b^
Dose reduction	
Absent	83 (69.7%)^b^
Present	36 (30.3%)^b^
Adverse events	
Grade 3-4	45 (37.8%)^b^
Grade 1-2	52 (43.7%)^b^
Absent	22 (18.5%)^b^

a: median (range); b: number (percentage); c: mean±standard deviation(SD); HBV: hepatitis B virus; HCV: hepatitis C virus; AFP: alpha-fetoprotein; LDH: Lactate dehydrogenase; ECOG PS: Eastern Cooperative Oncology Group performance status; TNM: Tumor-nodal-metastasis; BCLC: Barcelona Clinic Liver Cancer.

### The determination of the best cut-off of LDH

**Figure 2:**
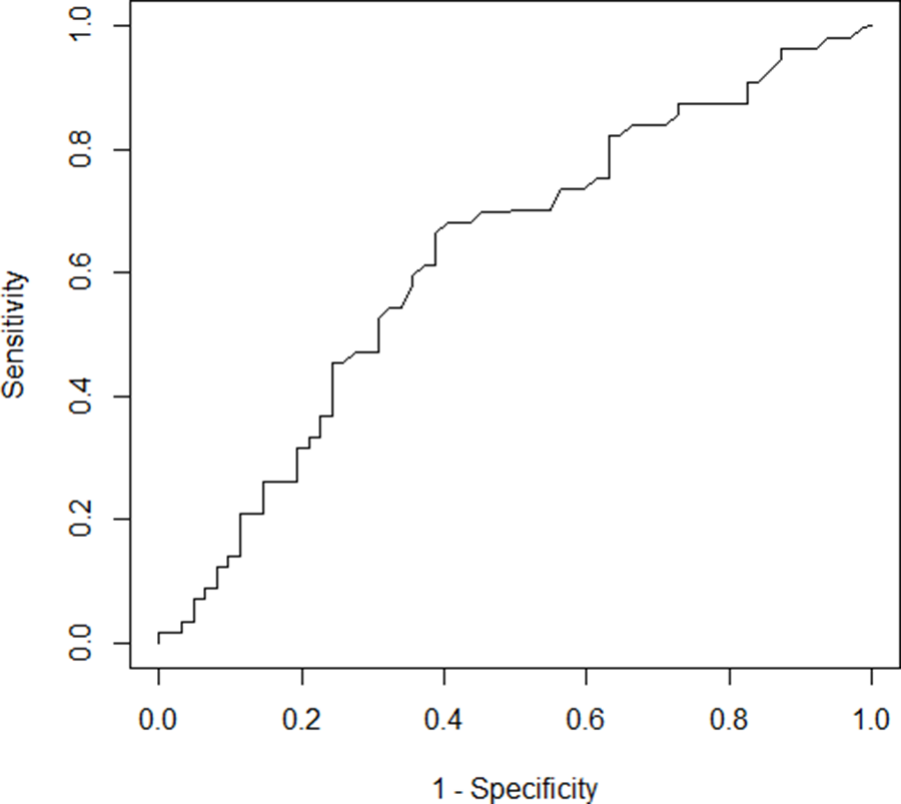
The determination of the best cut-off of pre-treatment lactate dehydrogenase (LDH) Time dependent receiver operating characteristic (ROC) curve for pre-treatment LDH as a predictor of patients died before the median overall survival as 14 months. The optimal threshold for pre-treatment LDH was 221 U/L. It resulted in sensitivity of 66.4% and a specificity of 61.3% (area under the ROC curve: 0.626).

The mean and median value of pre-treatment LDH in our study were 301.5 U/L and 223 U/L, respectively. In the follow-up, 91 patients died. The median OS and PFS of the patients were 14 (range, 2-73) months and 4 (range, 1-73) months, respectively. According to the time-dependent receiver operating characteristics (ROC) analysis (Figure 2) predicting patients died before the median overall survival, the optimal threshold for pre-treatment LDH was 221 U/L. It resulted in a sensitivity of 66.4% and a specificity of 61.3% (area under the ROC curve: 0.626, Figure 2). This threshold was used in further analyses. Thus 55 (55/119, 46.2%) patients were classified as the low pre-treatment LDH group and 64 (64/119, 53.8%) patients were classified as the high pre-treatment LDH group.

### The relationship between pre-treatment LDH and clinicopathological factors

When the patients were subdivided into the high pre-treatment LDH group and low pre-treatment LDH group, we found that patients in the high pre-treatment LDH group predisposed to be featured with lower serum albumin (*P*=0.034), presence of macroscopic vascular invasion (*P*<0.001), advanced Child-Pugh class (*P*=0.006), advanced T category (*P*=0.045), higher AFP (*P*=0.026), and higher serum total bilirubin (*P*<0.001) (Table 2).

**Table 2:** Comparison of clinicopathological features between patients in high pre-treatment LDH group (LDH ≥ 221 U/L) and low pre-treatment LDH group (LDH <221 U/L)

Variable	Value		*P*
	Low pre-treatment LDH (n=55)	High pre-treatment LDH (n=64)	
Age (years)	52.7±10.6^a^	54.8±10.7^a^	0.280
Gender (Male/Female)			0.559
Male	48(40.3%)^b^	58 (48.7%)^b^	
Female	7 (5.9%)^b^	6 (5.1%)^b^	
Albumin (g/L)	40.7±5.3^a^	38.6±5.3^a^	**0.034**
Total Bilirubin (μmol/L)	15.5±7.7^a^	28.0±18.7^a^	**<0.001**
Prothrombin time (seconds)	12.1±0.9^a^	12.2±1.2^a^	0.488
AFP (ng/ml)	9521.5±48440.7^a^	39527.4±92088.8^a^	**0.026**
Child-Pugh Class			**0.006**
A	54 (45.4%)^b^	53 (44.5%)^b^	
B	1 (0.9%)^b^	11 (9.2%)^b^	
Tumor number			0.945
Solitary	22 (18.5%)^b^	26 (21.9%)^b^	
Multiple	33 (27.7%)^b^	38 (31.9%)^b^	
Tumor size (cm)	6.3±3.7^a^	7.8±5.3^b^	0.091
Macrovascular invasion			**<0.001**
Absent	43 (36.1%)^b^	29 (24.4%)^b^	
Present	12 (10.1%)^b^	35 (29.4%)^b^	
ECOG			0.050
0	53 (44.5%)^b^	55 (46.2%)^b^	
1	2 (1.7%)^b^	9 (7.6%)^b^	
T category			**0.045**
1	18 (15.1%)^b^	12 (10.1%)^b^	
2	11 (9.2%)^b^	7 (5.9%)^b^	
3	26 (21.8%)^b^	43 (36.1%)^b^	
4	0 (0%)^b^	2 (1.7%)^b^	
N category			0.312
0	40 (33.6%)^b^	41 (34.5%)^b^	
1	15 (12.6%)^b^	23 (19.3%)^b^	
M category			0.720
0	25 (21.0%)^b^	27 (22.7%)^b^	
1	30 (25.2%)^b^	37 (31.1%)^b^	
TNM			0.439
II	7 (5.9%)^b^	4 (3.4%)^b^	
III	15 (12.6%)^b^	21 (17.6%)^b^	
IV	33 (27.7%)^b^	39 (32.8%)^b^	
BCLC stage			0.252
B	9 (7.6%)^b^	6 (5.0%)^b^	
C	46 (38.7%)^b^	58 (48.7%)^b^	
Previous treatments			0.234
Absent	6 (5.0%)^b^	12 (10.1%)^b^	
Present	49 (41.2%)^b^	52 (43.7%)^b^	
Concurrent treatments			0.786
Absent	21 (17.6%)^b^	26 (21.9%)^b^	
Present	34 (28.6%)^b^	38 (31.9%)^b^	
Discontinuation			0.124
Absent	37 (31.1%)^b^	51 (42.9%)^b^	
Present	18 (15.1%)^b^	13 (10.9%)^b^	
Dose reduction			0.512
Absent	40 (33.6%)^b^	43 (36.1%)^b^	
Present	15 (12.6%)^b^	21 (17.6%)^b^	
Adverse events			0.338
Grade 3-4	18 (15.1%)^b^	27 (22.7%)^b^	
Grade 1-2	24 (20.2%)^b^	28 (23.5%)^b^	
Absent	13 (10.9%)^b^	9 (7.6%)^b^	

N: number; a: mean±standard deviation(SD); b: number (percentage); HBV: hepatitis B virus; HCV: hepatitis C virus; AFP: alpha-fetoprotein; LDH: lactate dehydrogenase; ECOG PS: Eastern Cooperative Oncology Group performance status; TNM: Tumor-nodal-metastasis; BCLC: Barcelona Clinic Liver Cancer.

Significant results were expressed in bold.

### Pre-treatment LDH and survival outcomes

**Figure 3:**
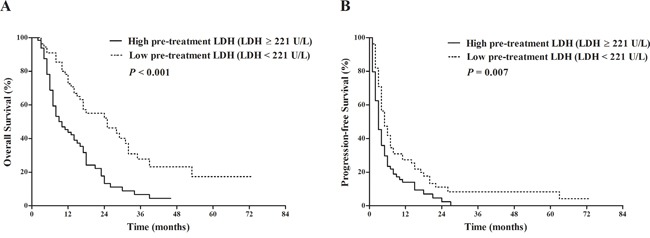
Comparison of survival outcomes between patients with pre-treatment LDH≥ 221 U/L vs. pre-treatment LDH < 221 U/L Kaplan-Meier survival analysis of overall survival (OS) **A**. and progression-free survival (PFS) **B**. LDH ≥ 221 U/L (64 patients) vs. LDH < 221 U/L (55 patients): median OS 9 vs. 25 months, *P* < 0.001; median PFS 3 vs. 5 months, *P* = 0.007.

Univariate analysis revealed that presence of macroscopic vascular invasion (*P*<0.001), higher pre-treatment LDH level (*P*<0.001) (Figure 3A), higher AFP level (*P*=0.001), advanced Child-Pugh class (*P*=0.004), larger tumor size (*P*<0.001), advanced T category (*P*<0.001), presence of concomitant treatment (*P*=0.001), presence of dose reduction (*P*=0.045) and advanced BCLC stage (*P*=0.032) were significantly associated with the OS (Table 3). The subsequent multivariate analysis found that higher pre-treatment LDH level (hazard ratio (HR) = 2.174, 95% confidence interval (CI) : 1.316-3.593, *P*=0.002), larger tumor size (HR=2.010, 95% CI: 1.176-3.435, *P*=0.011) and presence of concomitant treatments (HR=0.460, 95% CI: 0.287-0.738, *P*=0.001) were the independent prognostic factors for OS (Table 3). Regarding the PFS, advanced BCLC stage (*P*=0.010), advanced TNM stage (*P*=0.021), presence of extrahepatic metastasis (*P*=0.009), larger tumor size (*P*=0.011) and higher pre-treatment LDH level (*P*=0.007) (Figure 3B) gained statistical significance in the univariate analysis (Table 4). Higher pre-treatment LDH (HR=1.535, 95% CI: 1.045-2.255, *P*=0.029) and larger tumor size (HR=1.801, 95% CI: 1.170-2.772, *P*=0.008) were identified as the independent prognostic factors for PFS (Table 4).

**Table 3:** Univariate and multivariate analysis of the overall survival

Variable	N	Median OS (months)	Univariate analysis	Multivariate analysis		
			*P*	HR	95% CI	*P*
Age (years)			0.917			
≥ 60	38	16				
< 60	81	14				
Gender			0.145			
Male	106	17				
Female	13	11				
Albumin (g/L)			0.927			
≥ 35	94	16				
< 35	25	14				
Total Bilirubin (μmol/L)			0.102			
≥ 17.1	63	12				
< 17.1	56	18				
Prothrombin time (seconds)			0.124			
≥ 13	26	10				
< 13	93	18				
Pre-treatment LDH (U/L)			**<0.001**	**2.174**	**1.316-3.593**	**0.002**
≥ 221	64	9				
< 221	55	25				
AFP (ng/ml)			**0.001**	1.584	0.955-2.627	0.075
≥ 20	78	13				
< 20	41	23				
Child-Pugh Class			**0.004**	1.464	0.664-3.228	0.345
A	107	17				
B	12	7				
Tumor number			0.347			
Solitary	48	16				
Multiple	71	14				
Tumor size (cm)			**<0.001**	**2.010**	**1.176-3.435**	**0.011**
≥ 5	60	11				
< 5	59	23				
Macrovascular invasion			**<0.001**	0.919	0.487-1.733	0.794
Absent	72	21				
Present	47	10				
ECOG PS			0.220			
0	108	15				
1	11	16				
T category			**<0.001**	1.117	0.569-2.194	0.748
1-2	48	24				
3-4	71	11				
N category			0.745			
0	81	17				
1	38	12				
M category			0.878			
0	52	15				
1	67	16				
TNM						
II-III	47	15	0.963			
IV	72	16				
BCLC stage			**0.032**	1.399	0.656-2.987	0.385
B	15	29				
C	104	14				
Previous treatments			0.068			
Absent	18	8				
Present	101	16				
Concomitant treatments			**0.001**	**0.460**	**0.287-0.738**	**0.001**
Absent	47	9				
Present	72	18				
Discontinuation			0.768			
Absent	88	15				
Present	31	17				
Dose reduction			**0.045**	1.292	0.758-2.200	0.256
Absent	83	17				
Present	36	11				
Adverse events			0.195			
Grade 3-4	45	13				
Grade 1-2	52	18				
Absent	22	15				

N: number; HBV: hepatitis B virus; HCV: hepatitis C virus; AFP: alpha-fetoprotein; LDH: lactate dehydrogenase; ECOG PS: Eastern Cooperative Oncology Group performance status; BCLC: Barcelona Clinic Liver Cancer.

Significant results were expressed in bold.

**Table 4:** Univariate and multivariate analysis of the progression-free survival

Variable	N	Median PFS (months)	Univariate analysis	Multivariate analysis		
			*P*	HR	95% CI	*P*
Age (years)			0.648			
≥ 60	38	4				
< 60	81	4				
Gender			0.218			
Male	106	4				
Female	13	3				
Albumin (g/L)			0.967			
≥ 35	94	4				
< 35	25	4				
Total Bilirubin (μmol/L)			0.482			
≥ 17.1	63	4				
< 17.1	56	4				
Prothrombin time (seconds)			0.846			
≥ 13	26	4				
< 13	93	4				
Pre-treatment LDH (U/L)			**0.007**	**1.535**	**1.045-2.255**	**0.029**
≥ 221	64	3				
< 221	55	5				
AFP (ng/ml)			0.066			
≥ 20	78	3				
< 20	41	5				
Child-Pugh Class			0.538			
A	107	4				
B	12	4				
Tumor number			0.384			
Solitary	48	5				
Multiple	71	4				
Tumor size (cm)			**0.011**	**1.801**	**1.170-2.772**	**0.008**
≥ 5	60	3				
< 5	59	5				
Macrovascular invasion			0.348			
Absent	72	4				
Present	47	3				
ECOG PS			0.634			
0	108	4				
1	11	5				
T category			0.284			
1-2	48	5				
3-4	71	4				
N category			0.101			
0	81	4				
1	38	4				
M category			**0.009**	1.691	0.691-4.141	0.250
0	52	5				
1	67	3				
TNM			**0.021**	1.041	0.415-2.612	0.932
II-III	47	6				
IV	72	4				
BCLC stage			**0.010**	1.224	0.597-2.510	0.582
B	15	8				
C	104	4				
Previous treatments			0.989			
Absent	18	3				
Present	101	4				
Concomitant treatments			0.052			
Absent	47	3				
Present	72	5				
Discontinuation			0.940			
Absent	88	4				
Present	31	5				
Dose reduction			0.751			
Absent	83	4				
Present	36	4				
Adverse events			0.656			
Grade 3-4	45	4				
Grade 1-2	52	4				
Absent	22	3				

N: number; HBV: hepatitis B virus; HCV: hepatitis C virus; AFP: alpha-fetoprotein; LDH: lactate dehydrogenase; ECOG PS: Eastern Cooperative Oncology Group performance status; BCLC: Barcelona Clinic Liver Cancer.

Significant results were expressed in bold.

### The prognostic value of pre-treatment LDH in BCLC stage C patients

**Figure 4:**
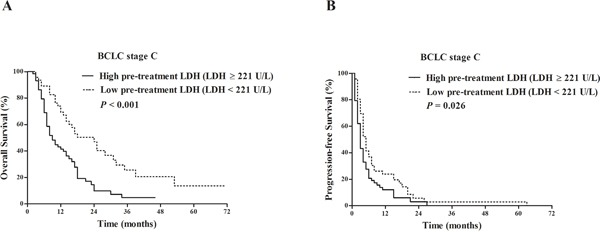
Comparison of survival outcomes between patients with pre-treatment LDH≥ 221 U/L vs. pre-treatment LDH < 221 U/L in BCLC stage C subgroup Kaplan-Meier survival analysis of overall survival (OS) **A**. and progression-free survival (PFS) **B**. LDH ≥ 221 U/L (58 patients) vs. LDH < 221 U/L (46 patients): median OS 9 vs. 24 months, *P* < 0.001; median PFS 3 vs. 5 months, *P* = 0.026.

For the 104 patients at BCLC stage C, the median OS and PFS of patients with pre-treatment LDH ≥ 221 U/L were 9 months and 3 months, respectively, which were significantly shorter than those of patients with pre-treatment LDH < 221U/L as 24 months (*P* < 0.001) and 5 months (*P*=0.026), respectively (Figure 4A&4B).

### The prognostic value of ΔLDH during the treatment

**Figure 5:**
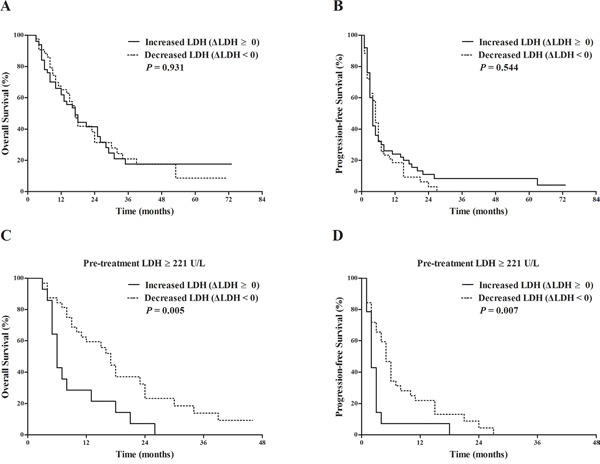
Comparison of survival outcomes between patients according to ΔLDH (variance between pre-treatment LDH and LDH level after 3 months of sorafenib treatment), which were available in 93 patients Kaplan-Meier survival analysis of overall survival (OS) **A**. and progression-free survival (PFS) **B**. according to ΔLDH. ΔLDH ≥ 0 (50 patients) vs. ΔLDH < 0 (43 patients): median OS 17 vs. 17 months, *P* = 0.931; median PFS 4 vs. 5 months, *P* = 0.544. Kaplan-Meier survival analysis of overall survival (OS) **C**. and progression-free survival (PFS) **D**. according to ΔLDH in patients with high pre-treatment serum lactate dehydrogenase (LDH ≥ 221 U/L). ΔLDH ≥ 0 (14 patients) vs. ΔLDH < 0 (32 patients): median OS 6 vs. 17 months, *P* = 0.005; median PFS 2 vs. 5 months, *P* = 0.007.

As the median PFS was 4 months in our study, we evaluated the variance between pre-treatment LDH and LDH level after 3 months of sorafenib treatment (ΔLDH). The relevant data were available in 93 patients. The median of ΔLDH was 0 with a range from -1331 to 1034. Fifty patients had increased LDH level while the LDH level decreased in the rest 43 patients. Patients whose ΔLDH ≥ 0 did not have significant different OS and PFS compared with those of patients whose ΔLDH < 0 (median OS: 17 months vs. 17 months, *P*=0.931; median PFS: 4 months vs. 5 months, *P*=0.544. Figure 5A&5B). We then subdivided the patients according to their pre-treatment LDH level. For the patients whose pre-treatment LDH ≥ 221 U/L, the median OS and PFS of the patients whose ΔLDH ≥ 0 were 6 months and 2 months, respectively, which were significantly shorter than those of patients whose ΔLDH < 0 as 17 months (*P*=0.005) and 5 months (*P*=0.007), respectively (Figure 5C&5D).

### Subgroup analyses

**Figure 6:**
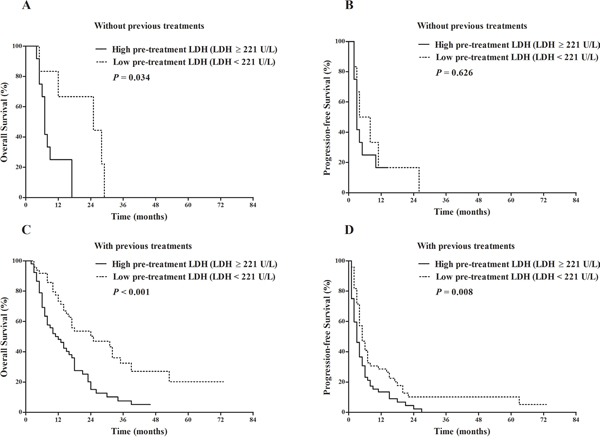
Comparison of survival outcomes between patients with pre-treatment LDH ≥ 221 U/L vs. pre-treatment LDH < 221 U/L stratified by the presence/absence of previous treatments Kaplan-Meier survival analysis of overall survival (OS) **A**. and progression-free survival (PFS) **B**. in patients who did not have previous treatments. LDH ≥ 221 U/L (12 patients) vs. LDH < 221 U/L (6 patients): median OS 7 vs. 25 months, *P* = 0.034; median PFS 3 vs. 6 months, *P* = 0.626. Kaplan-Meier survival analysis of overall survival (OS) **C**. and progression-free survival (PFS) **D**. in patients who had previous treatments. LDH ≥ 221 U/L (52 patients) vs. LDH < 221 U/L (49 patients): median OS 11.5 vs. 25 months, *P* < 0.001; median PFS 3 vs. 5 months, *P* = 0.008.

**Figure 7:**
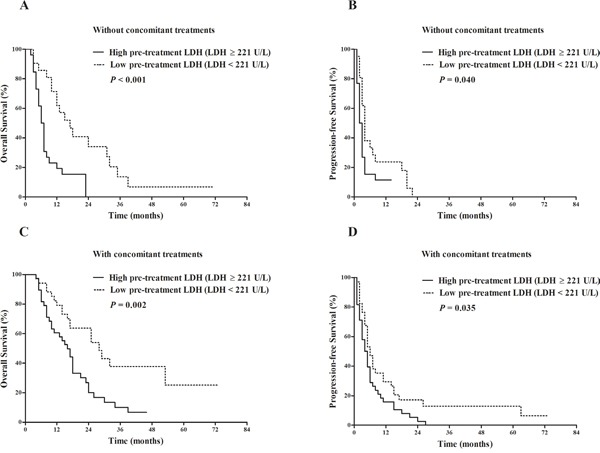
Comparison of survival outcomes between patients with pre-treatment LDH ≥ 221 U/L vs. pre-treatment LDH < 221 U/L stratified by the presence/absence of concomitant treatments Kaplan-Meier survival analysis of overall survival (OS) **A**. and progression-free survival (PFS) **B**. in patients who did not have concomitant treatments. LDH ≥ 221 U/L (26 patients) vs. LDH < 221 U/L (21 patients): median OS 6.5 vs. 17 months, *P* < 0.001; median PFS 2.5 vs. 4 months, *P* = 0.040. Kaplan-Meier survival analysis of overall survival (OS) **C**. and progression-free survival (PFS) **D**. in patients who had concomitant treatments. LDH ≥ 221 U/L (38 patients) vs. LDH < 221 U/L (34 patients): median OS 16 vs. 28 months, *P* = 0.002; median PFS 4.5 vs. 6 months, *P* = 0.035.

We further evaluated the prognostic effects of LDH on OS and PFS according to the presence/absence of previous treatments and presence/absence of concomitant treatments. Except for the subgroup analyzing the relationship between LDH and PFS in patients without previous treatments that only contained 18 patients (Figure 6B), the rest subgroup analyses did not undermine the prognostic value of LDH (Figures 6–7).

## DISCUSSION

The present retrospective analysis, to the best of our knowledge, is the first study systematically evaluating the prognostic value of serum LDH in HCC patients treated by sorafenib from the HBV endemic area. Our results showed that elevated pre-treatment serum LDH might be an indicator of decreased OS and decreased PFS. Moreover, we evaluated the prognostic value of the variance of LDH level during the treatment. In the subgroup of patients with high pre-treatment LDH level, we found that increased LDH level after 3 months of sorafenib treatment predicted poor survival outcomes. Our results suggested that serum LDH might be a prognostic indicator for HCC patients treated by sorafenib. And the alternation of serum LDH during the treatment should be monitored for patients with high pre-treatment LDH level.

Chiefly based on 2 RCTs, i.e. the SHARP trial [21] and Oriental trial [20], sorafenib has been widely accepted as the standard treatment for patients with advanced HCCs. Remarkable variances in the patients’ clinical outcomes remain a major concern of the sorafenib treatment [8]. In contrast to several other molecular targeted regiments such as cetuximab and imatinib, no widely accepted biomarker predicting the clinical efficacy of sorafenib has been identified yet [22]. The indication of the sorafenib treatment is still based on the clinical parameters such as the tumor staging, liver function reserve and performance status [5], which calls for more specific criteria for selecting the patients who may benefit from sorafenib treatment.

LDH is required for tumor maintenance, progression, and metastasis [23]. Cancer cells maintain high aerobic glycolytic rates and produce high levels of lactate and pyruvate, even under the oxygen-rich environment, which is also known as the “Warburg effect” [24]. LDH is a key enzyme in the conversion of pyruvate to lactate under anaerobic conditions [9]. The Warburg effect may stimulate the secretion of LDH; and the increased level of LDH may in turn amplify the Warburg effect [25]. Hypoxia in the tumor microenvironment, which may be partially accompanied by the elevated LDH level, is sufficient to activate HIF-dependent expression of several downstream genes [10]. These include genes encoding for vascular endothelial growth factor, erythropoietin and many enzymes involved in angiogenesis and cellular metabolism, which can further modulate the tumor development and confer treatment resistance [10, 26]. Previous studies have demonstrated that pretreatment serum LDH level could predict the clinical outcomes of HCC patients receiving TACE treatment [16]. And its prognostic value in HCC patients treated by sorafenib have been proved in 2 Italian studies in which HCV-related HCC predominated [17, 18]. Our study indicated that LDH was a prognostic factor in HBV-related HCC patients from a Chinese cohort for the first time.

In our study, significant associations between pre-treatment serum LDH level and several adverse clinicopathological factors were detected. Increased AFP level and advanced T category have long been regarded as indicators of tumor burden as well as the aggressive behavior of the HCC [27]. Thus it was quite possible that increased LDH level was accompanied by the elevation of AFP level and advanced T category, as the augment of tumor burden might upgrade the anaerobic metabolism of the tumor tissue. Patients with macroscopic vascular invasion predisposed to have higher pretreatment LDH level, which might be partially illustrated by the hypoxia secondary to decreased blood supply. As LDH is also taken as a serum biomarker of liver cell damage in the liver function test, it was quite understandable that higher LDH level might be closely associated with advanced Child-Pugh class, lower serum albumin and elevated serum bilirubin level.

The prognostic value of LDH in sorafenib treated HCC patients has been studied in the Italian population, where HCV-related HCC predominates. The HBV-related HCC differs from the HCV-related HCC a lot [19]. HBV is usually transmitted vertically while HCV is usually transmitted horizontally. Thus HBV-related HCC patients are relatively younger and are more likely to have heavier tumor burden when compared with HCV-related ones [28]. It was observed that the median OS and time to progress (TTP) of the patients in the Oriental study [20], which was conducted in the Asian-Pacific region where HBV-related HCC predominated, were shorter than those of the patients in the globally performed SHARP study [21] (median OS: 6.5 months vs. 10.7 months; median TTP : 2.8 months vs. 5.5 months). An unplanned retrospective analysis of SHARP study also showed that the median OS was 14 months in the HCV-related HCC subgroup, which was longer than that of the overall patients as 10.9 months [28]. These suggested that sorafenib might be more efficacious in HCV-related HCC. These findings could be partially explained by the fact that Raf-1, a kinase involved in the HCC development that could be upregulated by the HCV core proteins, expressed differently between HCV and HBV related HCC patients [29]. As Raf-1 is one of the targets of sorafenib [30], its varied expression may result in varied clinical outcomes. Given that the prognostic value of LDH has been previously studied in HCV endemic region [17, 18], it is quite essential to evaluate its prognostic value in HBV endemic area.

Generally, the tumor burden is much more severe in HCC patients from China when compared with the patients from Western countries [3, 31]. The survival benefit by sorafenib in the Oriental study was less than that in the SHARP study (median OS: 6.5 months vs. 10.7 months, median TTP: 2.8 months vs. 5.5 months), despite the 2 trials adopted the same patient entry criteria. This issue could be partially explained by the fact that patients enrolled in the Oriental trial were generally at worse performance status and more advanced stage of disease at the initiation of sorafenib therapy than those of the patients in the SHARP study [20, 21, 31]. Besides, differences in some other demographic features, e.g., age of onset, gender distribution and change of incidence rate over time for HCC between China and Western developed countries were also observed [3, 31]. Thus it would be of novelty to perform the present study, as it provided additional information about the prognostic value of LDH in Chinese HBV-related HCC patients who generally bear greater tumor burden and worse performance status.

In addition, the cut-off value defining elevated pre-treatment serum LDH level was not consistent among the reported studies. Faloppi et al. [17] set it as 407 U/L and Sacco et al. [18] set is as 297 U/L; while our study determined it as 221 U/L through the time dependent ROC analysis. The discrepancy could be partially explained by the limited sample size and the differences in ethnical background and etiologies for the underlying chronic liver diseases as well. Therefore more relevant clinical evidence is needed to reach a uniform applied cut-off value.

As the studied patients were from the real-life clinical practice, part of the patients were previously treated by loco regional therapies. And also some patients underwent concomitant treatments after the initiation of sorafenib treatments. Presence of concomitant treatment was significantly related with prolonged OS in our study, which could be partially explained by the fact that patients who received concomitant treatments were more likely to have less serious tumor burden and relatively better liver function reserve compared with those who received sorafenib monotherapy. Besides, majority of the subgroup analyses stratified by the presence/absence of previous treatments and presence/absence of concomitant treatments did not undermine the prognostic value of LDH (Figures 6–7), which further upheld the steadiness of our results.

The strength of the present study came as that it involved the largest sample size to date and was the first report from the HBV endemic area so far. LDH is a commonly used serum biomarker, which is easy and cheap to detect and, thus, appropriate for the use in routine clinical practice [11]. Admittedly, there were some limitations in our study. First of all, as the study was retrospectively performed, it was susceptible to several biases such as the selection bias and recalling bias. Exploring the relationship between LDH and other metabolic markers might further strengthen the prognostic power of LDH. Several widely known “metabolic markers”, such as albumin, total bilirubin, AFP and so on, have been taken into the analysis. Meanwhile we could not get access to the data of more metabolic markers due to the retrospective nature of the study. In the future, we will perform studies with reasonable study design to evaluate the relationship between LDH and more metabolic markers, as well as further validate the prognostic value of LDH in HBV related HCC patients treated by sorafenib. Secondly, the alternation of serum LDH level during the treatment was not available in 26 patients due to the retrospective nature of the study. Thirdly, it was known that LDH had 5 isoenzymes, and each of them might function differently in the tumor progression. In the present study, we could not get access to the data of the serum level of the isoforms, which called for more detailed researches in the future. Additionally, many clinicopathological factors such as distal metastasis did not turn to be statistical significant in the survival analysis. The loss of significant relationship between the factors and survival outcomes might be partially attributed to the relatively small sample size in the present study.

## CONCLUSIONS

LDH, which is closely related with the glycolytic and angiogenetic process of malignant tumor, appears to be a prognostic factor for patients with HCC receiving sorafenib treatment. Further relevant studies are warranted before serum LDH can be used as a routine index predicting the clinical efficacy of sorafenib in HCC patients.

## MATERIALS AND METHODS

### Patients selection

From January 2010 to December 2014, 176 consecutive patients with advanced HCC received sorafenib treatment in our hospital. The medical records and follow up information were reviewed. Inclusion criteria for this study were: (1) diagnosis of HCC was confirmed by pathological examination or was based on the American Association for the Study of Liver Diseases (AASLD) practice guideline [5]; (2) HCCs at advanced stage not eligible for liver resection or refractory to loco regional treatments such as RFA, percutaneous ethanol injection (PEI), and TACE. And the last session of the loco regional treatments must have been stopped at least 4 weeks before the initiation of sorafenib treatment; (3) Barcelona Clinic Liver Cancer (BCLC) stage B or C; (4) continuous administration of sorafenib ≥ 1 month, (5) performance status of 0 or 1 according to Eastern Cooperative Oncology Group performance status (ECOG PS) classification; (6) Child-Pugh class A or B. The exclusion criteria were as follows: (1) patients with ECOG PS score greater than 2; (2) patients at BCLC stage A or D; (3) patients with insufficient data; (4) patients with HCV-related HCC; (5) patients who had comorbidities including injury, cardiac disease, secondary primary malignancy, hypothyroidism, gastrointestinal bleeding up to 4 weeks before the initiation of sorafenib treatment and anemia were excluded; (6) patients lost to follow up. Finally 119 patients were taken into the retrospective analysis (Figure 1). All patients received sorafenib with standard schedule (400 mg bid continuously) at initiation. The treatment was continued until disease progression and (or) development of intolerable toxicity. Dose reduction was applied as clinically indicated. Patients’ informed consent was not required owing to the retrospective nature of the study. The primary end point of the study was OS. The secondary end point was PFS.

### Serum LDH level examination

Pre-treatment LDH serum levels were tested with the blood sample collected within 1 month prior the initiation of sorafenib treatment. The procedure followed the International Federation of Clinical Chemistry and Laboratory Medicine (IFCC) method. Serum LDH level was determined by LDH test reagent (Lactate Dehydrogenase acc. to IFCC ver.2 (LDHI2), Roche, Germany) using Roche cobas^®^ 8000 automatic biochemical analyzer within 2 hours after sample collection.

### Data collection

Clinical data including patient demographics (e.g. age, gender), etiology of underlying liver disease, previous anti-cancer treatments, serum biochemical test, serum LDH level, serum AFP level, tumor number, tumor size, and ECOG PS were obtained from patients’ medical records. Tumor staging was graded according to the Barcelona Clinic Liver Cancer (BCLC) staging system [32].

### Follow up

The last date of follow-up was December 31^st^, 2015. The follow-up were performed through face-to-face or telephone interview every 3 months or when tumor recurrence was highly suspected. At each visit, the information of physical examination, serum biochemistry test, chest radiography, abdominal ultrasonography (US), contrast-enhanced computer tomography (CT) scans, and (or) liver magnetic resonance imaging (MRI) was collected. The side effects of sorafenib treatment were also interviewed. Two radiologists independently evaluated the response to sorafenib treatment every 12 weeks after the initiation of sorafenib therapy by modified Response Evaluation Criteria in Solid Tumors (mRECIST) [33]. When disagreement occurred, a senior oncologist would be referred. Sorafenib treatment continued until disease progression and (or) unacceptable drug-related toxicity. Toxicity grade was assessed using the National Cancer Institute Common Toxicity Criteria for Adverse Events (version 3.0).

### Definitions

Overall survival time of patients was calculated from the date of initiation of sorafenib treatment to the date of last follow-up or death. Progression-free survival was defined as the time duration between the initiation of sorafenib treatment to the date of last follow-up or tumor progression. Tumor size referred to the size of the largest tumor lesion in case that multiple lesions existed. Patients were divided into two subgroups according to the best cut-off value for pre-treatment LDH determined by the time dependent receiver operating characteristics (ROC) curve analysis for the overall survival [34]. ΔLDH denoted the variance between pre-treatment LDH and LDH level after 3 months of sorafenib treatment.

### Statistical methods

Continuous variables were expressed as means with standard deviations (SD) or medians with ranges. Categorical variables were expressed as frequencies with percentages. For group comparisons, Chi-square test or Fisher's exact test (categorical variables) and independent sample T-test (continuous variables) were used to compare the differences between subgroups. Univariate analysis for the OS and PFS was performed by the Kaplan–Meier method and the differences were analyzed by the log-rank tests. Significant factors identified in univariate analysis were subsequently enrolled in the multivariate Cox proportional hazard model. A two-tailed *P* < 0.05 was considered statistically significant. Time dependent ROC curve analysis was performed by R software version 2.15.1 (http://www.r-project.org). And the rest statistical analyses were performed using SPSS 11.5 for Windows (SPSS Inc, Chicago, IL).

## SUPPLEMENTARY TABLES



## Abbreviations

LDH: Lactate dehydrogenase
HCC: hepatocellular carcinoma
FDA: Food and Drug Administration
OS: overall survival
PFS: progression-free survival
HBV: hepatitis B virus
HCV: hepatitis C virus
AFP: alpha-fetoprotein
RFA: radiofrequency ablation
TACE: transcatheter hepatic arterial chemoembolization
BCLC: Barcelona Clinic Liver Cancer stage
AASLD: American Association for the Study of Liver Diseases
ECOG PS: Eastern Cooperative Oncology Group performance status
IFCC: International Federation of Clinical Chemistry and Laboratory Medicine
mRECIST: modified Response Evaluation Criteria in Solid Tumors
