# Supplementary material for: Lactate dehydrogenase is a prognostic indicator in patients with hepatocellular carcinoma treated by sorafenib: results from the real life practice in HBV endemic area
Source: Oncotarget. 2016 Nov 17;7(52):86630–47. doi: 10.18632/oncotarget.13428 (PMC5349941; doi:10.18632/oncotarget.13428)
Supplement: Supplementary file 1 [file oncotarget-07-86630-s001.pdf]

Lactate dehydrogenase is a prognostic indicator in patients with hepatocellular carcinoma treated by sorafenib: results from the real life practice in HBV endemic area

SUPPLEMENTARY TABLE

Supplementary Table S1: Detailed previous and concomitant treatment information of the 119 HBV-related HCC patients treated by sorafenib

Therapy	Number (Percentage)
Previous treatments	101 (84.9%)
TACE	88 (73.9%)
Liver resection	30 (25.2%)
Radiofrequency ablation	8 (6.7%)
External radiotherapy	8 (6.7%)
Concomitant treatments	72 (60.5%)
TACE	67 (56.3%)
Radiofrequency ablation	7 (5.9%)
External radiotherapy	6 (5.0%)

TACE: transarterial chemoembolization.
